# Peripheral Nerve Injury: Stem Cell Therapy and Peripheral Nerve Transfer

**DOI:** 10.3390/ijms17122101

**Published:** 2016-12-14

**Authors:** Robert Sullivan, Travis Dailey, Kelsey Duncan, Naomi Abel, Cesario V. Borlongan

**Affiliations:** Department of Neurosurgery and Brain Repair, University of South Florida College of Medicine, 12901 Bruce B. Downs Blvd., Tampa, FL 33612, USA; rsulliv1@health.usf.edu (R.S.); tdailey@health.usf.edu (T.D.); kduncan3@health.usf.edu (K.D.)

**Keywords:** regeneration, Schwann cells, nerve graft, axonal injury, combination therapy, growth factors

## Abstract

Peripheral nerve injury can lead to great morbidity in those afflicted, ranging from sensory loss, motor loss, chronic pain, or a combination of deficits. Over time, research has investigated neuronal molecular mechanisms implicated in nerve damage, classified nerve injury, and developed surgical techniques for treatment. Despite these advancements, full functional recovery remains less than ideal. In this review, we discuss historical aspects of peripheral nerve injury and introduce nerve transfer as a therapeutic option, as well as an adjunct therapy to transplantation of Schwann cells and their stem cell derivatives for repair of the damaged nerve. This review furthermore, will provide an elaborated discussion on the sources of Schwann cells, including sites to harvest their progenitor and stem cell lines. This reflects the accessibility to an additional, concurrent treatment approach with nerve transfers that, predicated on related research, may increase the efficacy of the current approach. We then discuss the experimental and clinical investigations of both Schwann cells and nerve transfer that are underway. Lastly, we provide the necessary consideration that these two lines of therapeutic approaches should not be exclusive, but conversely, should be pursued as a combined modality given their mutual role in peripheral nerve regeneration.

## 1. Introduction

With an annual incidence of approximating 13 to 23 per 100,000 persons per year [1,2,3], the consequences of peripheral nerve injuries are notoriously devastating and life-altering. The morbidity and unintended negative effects (both physical and psychological) of current treatment on patients’ lives necessitates the need for continued translational research to provide alternative and potentially more efficacious treatment modalities. Peripheral nerves are comprised of myelinated and umyelinated fibers, with myelinated nerves surrounded by the specialized Schwann cells to provide insulation. Injuries to these structures are typically secondary to sudden stretch of a limb, laceration, compression, or ischemia. The primary insult is thought to result from direct forces applied while secondary injury is due to subsequent vascular ischemic damage. Anatomic redundancy in nerve length precludes injury with common tension, however, when exceeded; injury occurs [4,5]. Concurrent vascular injury to the vasa nervorum may lead to a compressive hematoma resulting in nerve ischemia, thereby exacerbating the primary injury.

In 1943, Seddon introduced a classification system to describe nerve injury, which included descriptions of neurapraxia, axonotmesis, and neurotmesis [6]. In 1951, Sunderland expanded this classification of nerve injury into five categories [7]. Axonotmesis is expanded to three grades for a total of five grades (Table 1). Grade 4 and 5 require surgical intervention.

Although the classifications above are based on histology and correlate to specific injury models, most nerve lesions are of mixed pathology. A 6th degree nerve injury has been proposed to describe mixed pathology [8]. Endogenous repair follows injury but is limited to 12–18 months [8] due to loss of neuromuscular junction endplates and muscle fibrosis. Repair is not sustained thereafter. Thus, the degree of recovery is inversely proportional to the extent of the damage [8,9,10]. Outside of a year from the injury, recovery is less likely and the patient may experience loss of function and chronic pain. Surgical options may serve to realign damaged nerves, through approximation of the ends or interposition of a graft; however, these procedures may be limited by the location of injury and length of the graft required, consequently providing suboptimal recovery [2,10,11].

## 2. Endogenous Nerve Healing in Response to Injury

Following peripheral nerve injury, the distal and proximal segments to the lesion, surrounding neurons, and non-neural cells all respond. Initially, the neuronal cell body enlarges, Nissl bodies dissolve, and the nucleus migrates peripherally, inducing protein synthesis [12]. Wallerian degeneration then proceeds, within 10 to 14 days, with fragmentation of the nerve distal to the lesion, however, this process does not occur immediately following injury. Primate studies demonstrate intact axons for days following the insult that may still function to transmit electric potentials upon stimulation [13,14]. Once initiated, fragmentation proceeds rapidly, completed within hours due to intrinsic proteases [15,16,17]. Schwann cells and macrophages further facilitate this process.

Schwann cells serve a pivotal role in nerve repair in several aspects such as degeneration, remyelination, and axonal growth. Their role begins with activation after the injury occurs. They then regress to a more primitive phenotype while upregulating growth-related genes that include neurotrophic and transcription factors that stimulate axonal growth [2,18,19]. Following axonal injury, the tip of the injured axon comes into contact with the intracellular contents of nearby Schwann cells and axons, as well as with inflamed neural tissue. Numerous trophic and growth-inducing molecules are secreted from these surrounding cells, resulting in a signaling cascade that begins with the release of ciliary neurotrophic factor by the damaged myelinated Schwann cells. Acute inflammatory changes at the site of the peripheral nerve lesion include an increase in local leukocytes that, along with the Schwann cells, help to clear the cell body of myelin debris [2,18,19]. The concomitant interaction of Schwann cells and surrounding environment promotes axonal sprouting, typically at a rate of 1 to 4 mm per day [2,20,21]. This endogenous repair yields a nerve with thinner myelin sheets, shorter nodal lengths, and decreased functional capacity [22]. The importance of Schwann cells in the regeneration phase of nerve repair is further underscored by studies demonstrating the crossing of significantly fewer axons into the distal area of injury following deletion of the gene for the gamma 1 laminin chain of peripheral Schwann cells [18,19,20,21,22]. When intervention is delayed, Schwann cells undergo apoptosis and the potential for repair diminishes.

## 3. Treatment for Peripheral Nerve Injury

The treatment for traumatic nerve injury is variable, ranging from conservative to surgical. Initial approaches may include early mobilization, physical therapy, and patient comfort. Alternatively, early surgical intervention may be favored to promote nerve regeneration. The timing of surgical intervention is dependent on whether the injury is open or closed. Closed injuries are often the result of stretch or crush injuries. Nerve injury occurs by demyelination, axonal loss, or a combination. When there is no clinical observation of improvement in a closed injury, an electrodiagnostic study is helpful to evaluate for neurapraxia and axonotmesis. Fourth and fifth degree injuries require surgical intervention [23]. The EDX study can differentiate these injuries, however, it only indirectly distinguishes between 3rd and 4th degree injuries. Nerve regeneration occurs in 2 ways, either by novel axonal regrowth or by collateral sprouting. The goal of an EDX study at 6 weeks is to localize the injury and assess for degree of nerve injury as well as make a determination of whether there is early reinnervation. If there is no clinical, improvement a repeat, study at 3 months may demonstrate evidence of early reinnervation by either novel axonal regrowth or collateral sprouting. If there is no evidence of either, surgical intervention is an option [23].

External neurolysis seeks to promote healing by isolating nerve from surrounding scarred tissue. More technical is the internal neurolysis procedure, which isolates conducting fascicles of nerves, and is recommended for axonotmetic lesions. This procedure can be advanced an additional step by end-to-end suturing of lesioned fascicles, termed split repair. If end-to-end approximation is not feasible then nerve grafting may be used to bridge the gap. This harvested nerve is interposed between the two free ends of the nerve and may be procured from several peripheral sites. Of note, algorithms for surgical intervention are described in the literature, but for the topic of this review, we will focus on another novel technique, nerve transfers, and the role Schwann cells may play in their efficacy.

Nerve transfer serves as a novel approach to rescue degenerative nerves following injury. The transfer, also known as neurotization, utilizes a nearby intact nerve and redirects it to the intact portion of the axon distal to the lesion. This technique effectively bypasses the region of the damaged nerve that may be impairing function. This procedure continues to gain traction as an alternative to nerve grafting, circumnavigating graft length limitations. The literature enumerates the potential nerve sites that can be transferred, such as the spinal accessory nerve, intercostal nerves, triceps nerve, ulnar nerve, and others [24].

Nerve repair is predicated on a host of factors, including the participation of Schwann cells. The rate of peripheral nerve regeneration is increased in the presence of endogenous Schwann cells. Thus, these cells may also serve a purposeful role in aiding in nerve repair following nerve transfer. Schwann cells have also demonstrated an enhanced response when associated with other cells, such as olfactory ensheathing cells (OEC). The literature provides evidence that these cells promote better myelination and repair to sciatic axon injury synergistically [25,26]. Further potential resides in the ability to modify Schwann cells, such as bioengineering adhesion molecules [27,28].

A review of the literature corroborates evidence that there is practicality for the use of stem cells to promote peripheral nerve repair [29]. Various stem cell lines have been investigated in repair during nerve grafts and conduits; however, their potential in nerve transfer must still be explored. Previous studies would suggest that Scwhann cells, and their progenitor cell line, will provide increased efficacy during nerve transfers, similarly to other treatment modalities. In this review, we will continue to define, explore the sources, discuss trials, and expand the potential beneficial role that stem cells and Schwann cells may serve in nerve transfer.

## 4. Sources of Schwann Cells

Neural crest cells provide the embryologic origin for Schwann cells [30,31]. The capacity of Schwann cells for proliferation, growth factor secretion, immune modulation, remyelination, and migration make them well suited for endeavors in neural repair, which have been reported in the literature for many years [32,33,34]. Functional recovery was evidenced in one such study, in which autologous Schwann cells in tubes were used to connect sciatic nerve stumps in rats [35]. This experimental method involving the use of transplanted Schwann cells in addition to axonal frameworks [36,37,38] avoids many of the drawbacks associated with surgical repair and can be accomplished using one of three different delivery systems: (1) a hollow tube filled with cells may be placed in the injured site; (2) the cells can be set in hydrogel prior to being packed into the tube lumen; or (3) the aforementioned cell-laden hydrogel can be loaded into a tube that contains a supportive scaffolding [39]. The first method of delivery is the most commonly studied; however, significant loss of cells is an important potential drawback [35,40,41,42]. The second [37,38] and third [36] techniques are associated with both better results as well as higher levels of cost and technical complexity. The risk of culture contamination by fibroblasts, possible harm to the donor nerve, and the large quantity of cells needed all restrict the efficacy and practicality of these methods [43] and have necessitated investigation into other sources of supportive cells.

It has been evidenced that differentiation of human NCSC into Schwann cells can be promoted with the use of synthetic matrix topography [44]. Cell alignment can be stimulated with the use of aligned fiber matrices comprised of electrospun fibers of varying alignments and diameters. In one study by Ren et al., the NCSCs that were most likely to develop a Schwann cell fate were those that used fiber matrices with average diameters between 600 nm and 1.6 μm [44]. Additionally, 2 weeks of predifferentiation in Schwann cell medium caused the human NCSC to be more responsive to the influence of aligned fiber topography than undifferentiated NCSC [44]. These results imply that a combination of the specified differentiation medium and the most favorable nanofiber matrix could be a very useful way to heavily influence NCSC differentiation into a Schwann cell lineage.

Although Schwann cells may be harvested from a variety of tissues, bone marrow mesenchymal cells, which have been investigated for many years within the realm of in vitro expansion and transplantation [45], are another potential source of support cells due to their availability, simplicity of retrieval, and associated safety. Mesenchymal stromal cells (MSC) are a type of multipotent somatic stem cell originating from a non-hematopoietic predecessor found in the bone marrow and are capable of differentiating into neural phenotypes, mesodermal cell lineages, and Schwann-like cells [46,47]. MSCs undergo transdifferentiation into a glial cell phenotype following exposure to suitable culture media, after which they express the proteins GFAP, p75, and S100 [46,47]. Cells differentiated in vitro have been shown to assist in functional and axonal recovery when coupled with artificial tubes and acellular grafts [48,49,50].

In addition to bone marrow and nervous tissue, skin, adipose, and olfactory tissues provide other potential cell sources. Skin precursor cells (SKPC), which are neural crest-related precursors found in the dermis, are capable of in vitro differentiation into neural crest cell types that display similar features to Schwann cells and peripheral neurons [51,52]. Furthermore, NCSC can be found in the bulge region of hair and whisker follicles. These can, in turn, provide a highly available supply of transplantable cells due to their ability to differentiate into Schwann-like cells. Functional improvement in rat models has been demonstrated in studies involving both NCSC and SKPC transplantation in spinal cord and peripheral nerve injuries [51,53,54,55].

In reference to adipose tissue, a variety of intrinsic neurotrophic factors are reportedly expressed by differentiated adipose-derived Schwann cells [56,57]. According to Reid et al., expression of anti-apoptotic m-RNA of Bcl-2 in the dorsal root ganglia was notably elevated following the addition of nerve-derived or adipocyte-derived Schwann cells into the location of repair [57]. Similarly, expression of pro-apoptotic m-RNA Bax and caspase-3 was reduced, resulting in some degree of neuroprotection [57]. One comparative study that focused on the use of various cell types within a fibrin tube for the repair of the sciatic nerve found that primary Schwann cells resulted in better outcomes than adipose or bone marrow-derived mesenchymal stem cells [58]. Nevertheless, some benefit to axonal restoration was achieved using adipose and bone marrow-derived mesenchymal stem cells when compared with the control group. Schwann cells derived from adipose tissue have also been employed in experimental models of facial palsy, with results including the electrophysiological and functional recovery of 0.8 cm facial nerve lesions in rats [59].

Olfactory epithelium may provide yet another source of Schwann cells for use in the treatment of peripheral nerve injury. A study by Ohnishi et al. demonstrated olfactory sphere (OS) cells generated in culture from rat olfactory mucosa expressed both astrocyte and oligodendrocyte progenitor cell (OPC) markers [60]. The OS cells differentiated into oligodendrocytes both in vitro and when they were grafted in vivo into the host tissue of injured rat spinal cords. However, when transected saphenous nerve endings were enclosed by silicone tubes containing collagen, the transplanted OS cells differentiated into Schwann cells, suggesting that the cells may have a higher affinity for a Schwann cell lineage in the context of peripheral nerve damage [60].

More recently, induced pluripotent stem cells (iPSC) have been given more attention for their promising role in tissue engineering and cell therapies [61,62]. One study used NCSCs derived from human iPSCs to form a bridge across transected sciatic nerves in a rat model. At one month, the NCSC-engrafted nerve conduits demonstrated accelerated regeneration compared to the ESC group and showed advanced axonal myelination [62]. Additionally, the cells derived from iPSCs had differentiated into Schwann cells and assimilated into the myelin sheath surrounding the axons, demonstrating the potential for the use of iPSC-derived Schwann cells in peripheral nerve injury.

When transplanted into the area of peripheral nerve damage, neural stem cells differentiate into Schwann-like cells [18]. These cells promote nerve regeneration, release neural growth factors, and reduce the need for immunosuppressants by augmenting the compatibility of transplanted and host tissues. Additionally, functional recovery within a spinal cord injury model is enhanced by the co-culture and transplantation of Schwann cells with neural stem cells by promoting the stem cells' differentiation into neurons [63].

## 5. Experimental Studies on Schwann-Like MSCs

A study of median nerve injuries in monkeys by Wakao et al. evidenced histological, electromyographical, and functional improvements by utilizing collagen nerve conduits packed with autologous bone marrow-derived MSC which were differentiated into Schwann cells in vitro [48]. Remarkably, another investigation focusing on ulnar nerve injuries in non-human primates showed that functional recovery was also achieved with the use of allogenic acellular nerves filled with autologous bone marrow MSCs that had not been differentiated [64]. The similarity in success rates between this study and studies using autografts and acellular nerves filled with autologous Schwann cells suggests that repair may be possible without pre-differentiation of the MSCs into Schwann cells prior to transplantation. Interestingly, an experiment involving the local transplantation of allogenic Schwann cells into a rat model of spinal cord injury resulted in the migration of endogenous Schwann cells to the injured area [65]. While this cannot yet be translated into peripheral nerve damage, it does give rise to the hypothesis of a paracrine effect whereby recovery is supported by stimulating the infiltration of nearby host cells into the site of injury, moderating the immune response, and increasing cell survival as opposed to pure replacement. However, a study using decellularized allogeneic arteries containing Schwann cells derived from adipose tissue for the treatment of facial nerve injuries in rats found that the grafted cells lived over 8 weeks following transplantation [66].

Schwann cells may exist in un-differentiated, differentiated, and de-differentiated states, and have the fate plasticity to transition from one state to another depending on conditions [67,68]. It is possible to maintain Schwann cells in a given state during culture, and indeed some literature supports the ability to influence the fate of these cells even following transplantation through such means as fibrin regulation [69]. However, it must be noted that it is possible that the differentiation states and fate maintenance of Schwann cells in culture and following transplantation may interfere with the therapeutic effects of cell-based therapy when using the cultured cells as the source of Schwann cells.

## 6. Clinical Trials of Novel Therapies in Peripheral Nerve Injury

While many studies have investigated the benefits of cell therapies in animal models of peripheral nerve injury, clinical applications of these technologies in the form of randomized controlled trials continue to be elusive. One such study conducted by the University of Stanford investigated the use of scaffolding tubes containing autologous cultured Schwann cells for the repair of short and long gaps in peripheral nerve injuries. However, as of yet no clinical data from this study has been reported in the literature. One study currently recruiting participants is investigating the safety of autologous human Schwann cells in patients with subacute spinal cord injury (ClinicalTrials.gov identifier NCT01739023). A similar study is evaluating the safety of local administration of autologous bone marrow stromal cells in chronic paraplegia (ClinicalTrials.gov identifier NCT01909154). However, these studies employ cell-based therapy for the treatment of central lesions rather than focusing only on peripheral nerve injuries.

Clinical trials involving conventional nerve transfer techniques without the adjunct of cell therapies are more numerous. One such study piloted by the Washington University School of Medicine is currently enrolling patients to investigate the efficacy of nerve transfer in restoring upper extremity function following cervical spine injury (ClinicalTrials.gov identifier NCT01899664). Another study currently underway from the Lawson Health Research Institute is looking at the benefits of distal nerve transfers in the treatment of severe compressive ulnar nerve injuries (ClinicalTrials.gov identifier NCT02281656). Existing clinical data shows that nerve transfer procedures lead to functional motor and sensory recovery in several types of peripheral nerve injury. However, the application of cell therapies in combination with nerve transfer has not been studied and represents an area of potential clinical interest.

## 7. Building a Case for Combined Peripheral Nerve Transfer and Cell Therapy

Peripheral nerve injuries frequently produce functional disability in young and productive individuals. Spontaneous peripheral nerve regeneration is a frustratingly slow process, occurring at a rate of approximately 1 mm per day, and is a function of multiple factors including degree of injury, time, distance, scar formation, end organ atrophy, and distal nerve degeneration. Without intervention, many patients would be left with some degree of functional disability significantly affecting quality of life. As mentioned previously, there are several available methods for the treatment of peripheral nerve injury, but nerve transfer is emerging as the preferred modality in many types of nerve injury.

Nerve transfers were originally described in the setting of brachial plexus injuries and this remains the area in which they are most often utilized. In adult upper brachial plexus injuries, nerve transfers were found to be more effective at restoring elbow flexion and shoulder abduction when compared to nerve grafting [70]. Recently, the use of nerve transfers has been widened to include repair of cervical spine injuries, ulnar and radial nerve injuries in the upper extremity, femoral nerve injuries in the lower extremity, and many more [71,72,73,74]. Current indications for nerve transfer include proximal nerve root avulsion, high peripheral nerve injuries, trauma with significant scarring at site of injury, and with large neuromas in-continuity and/or with multilevel injuries [75]. However, as our understanding of nerve topography grows, new transfers continue to be developed and we can expect these indications to broaden in the future.

Cell-based therapy is a promising branch of regenerative medicine. The idea of using cultured Schwann cells or bone marrow mesenchymal cells in conjunction with nerve transfer procedures may be an attractive alternative to more aggressive therapies. If successful, the treatment may lead to functional improvement as well as shortened recovery times, avoiding the hurdles of additional surgeries. Although the regenerative potential and the properties of the cells change slightly from tissue to tissue, the bone marrow still stands as a preferred source of hematopoietic and mesenchymal cells due safety and established clinical use, despite greater ease of harvest with other tissue types including skin and fat [45]. The use of allogeneic bone marrow-derived MSCs as an adjunct to nerve transfer for repair of peripheral nerve injury would be proposed as an alternative to the standard autologous nerve graft implant. The acute nature of many peripheral nerve injuries necessitates a readily available source of cells. MSCs are immunoprivileged and therefore, not readily rejected by the host, making them an ideal source for “off-the shelf” accessibility.

Cells would be harvested in advance, expanded in culture without further manipulation, tested for safety and quality, and then cryopreserved to ensure availability for clinical use. The necessary amount of cells would then be thawed and implanted around the neurotization site, ideally during the final stages of microsurgery. Immunosuppressive therapy would not be required, sequential functional and electrodiagnostic evaluations in the form of nerve conduction studies, and electromyography would be used to determine meaningful recovery. Either expected results would include repair and remyelination of axons as well as progressive functional improvement, through differentiation of the transplanted cells into Schwann-like cells or, more likely, through paracrine effects of the bone marrow-derived MSCs on the transferred nerve and remaining endogenous Schwann cells stimulating regeneration.

The application of MSCs to augment nerve transfer for repair of peripheral nerve injury may be associated with certain practical obstacles that would have to be navigated to ensure optimal results. The most obvious hurdle is cell viability after thawing and insertion into the surgical site. Reconstructive surgeries are often lengthy affairs and prolonged exposure of the cells to a hostile environment may reduce potential survival. Therefore, delay of cell transplantation until the final stages of the surgery, immediately followed by closure of the incision, may be critical to ensuring highest chances of cell survival. Care must be taken to avoid additional insults in the form of infection, and acidic or ischemic environments. Another concern is that MSC transplantation may lead to irregular, non-linear nerve growth patterns due to aberrant stimulation of axonal buds. This problem has been observed in nerves stimulated with growth factors, however, because of the direct end-to-end or end-to side union of donor and recipient nerve, aberrant sprouting is not expected following nerve transfer procedures augmented with MSCs. Finally, whenever stem cells are used there is a possibility that they may differentiate into lineages other than the one that is desired. Malignancy is not reported as a usual consequence of MSC administrations, but it is possible that due to the local host conditions the cells could differentiate into non-neural cell types. Therefore, clinical trials with adequate follow up would be needed to ensure the safety and efficacy of the proposed treatment.

In summary, despite a growing literature base illuminating the therapeutic benefits of cell-based therapy in experimental models of peripheral nerve injury, specific translational hurdles will need to be addressed before the realization of large clinical trials for this treatment. Moving forward, this form of cell therapy may evolve to include more advanced features such as the addition of trophic factors and manipulation of the cells for expression of neurotrophic factors or even adhesion molecules. To this end, pursuing cell therapy in combination with nerve transfer may allow improved outcomes by complementing each individual treatment’s weaknesses and strengths. Transplanted cells, via secreted trophic and paracrine factors, may aid in stimulating the regenerative capabilities of the transferred nerve. In tandem, the transferred nerve may close the physical gap of the injured peripheral nerve facilitating the survival of the transplanted cells, as well as providing the stem cells the ability to form appropriate nerve growth patterns and axonal projections.

## 8. Conclusions

Repair of peripheral nerve injury through spontaneous regeneration is often unsatisfactory even after microsurgical intervention, especially in the setting of severe injuries. Ensuing chronic pain and functional disability have been shown to affect victims of all ages, including those in an economically productive phase of their life. Nerve transfers are emerging as the preferred modality for treatment of several types of peripheral nerve injuries. Advantages include earlier muscle re-innervation, restoration of sensation, avoidance of surgery in scarred fields, and rare need for grafts. Despite the potential benefits, to our knowledge, there are currently no studies investigating the application of cell-based therapies as an adjunct to traditional nerve transfer procedures. Schwann cell cultures have demonstrated favorable results in the experimental models of peripheral nerve injury; however, the ideal source of cells has not yet been established. Bone marrow-derived mesenchymal cells present encouraging results and numerous advantageous properties including minimally invasive harvesting, high cell viability rates, immunoprivileged status, and the secretion of multiple trophic factors make them a possible front-runner for clinical translation.

Cultured cells, combined with adjunctive treatments such as nerve transfers may be a feasible approach for future clinical studies. The neurotropic effect of these cells at the transfer site, combined with a favorable effect over the surrounding environment, may offer shortened recovery times and improved functional restoration. Nerve transfers and cell-based therapy individually have evidence supporting their efficacy in the treatment of peripheral nerve injury; however, the marriage of these individually strong elements into one modality is a novel prospect that should be an exciting area of active research in the future.

## Figures and Tables

**Table 1:** Peripheral Nerve Injury Grading System.

Grade	Description
Grade I	-Neurapraxia, conduction block -Focal demyelination without any axonal degradation -Secondary to mild injury such as ischemia, compression, or toxins
Grade II	-Axonotmesis -The axon, in addition to the myelin sheath, is disrupted by irreversible damage -Neuronal stroma (endoneurium, perineurium, and epineurium) remains intact -Seen in crush, over-stretching, and percussion (such as blast or bullet) injuries -Nerve distal to the lesion undergoes Wallerian degeneration
Grade III	-Loss of axonal and endoneurial continuity
Grade IV	-Loss of axonal, endoneurial, and perineural continuity
Grade V	-Neurotemesis -The axon, myelin sheath, and stroma are all irreversibly damaged -Follows severe lesions such as laceration, percussion, or neurotoxins

## Abbreviations

EDX	electrodiagnostic
OEC	olfactory ensheathing cells
NCSC	neural crest stem cells
MSC	mesenchymal stromal cells
SKPC	skin precursor cells
OS	olfactory sphere
OPC	oligodendrocyte progenitor cell
iPSC	induced pluripotent stem cells
